# High Glycated Hemoglobin Instead of High Body Mass Index Might Increase the Urine *N*-Acetyl-β-D-glucosaminidase Con-Centration in Children and Adolescents with Diabetes Mellitus

**DOI:** 10.3390/life12060879

**Published:** 2022-06-12

**Authors:** Jin-Soon Suh, Kyoung Soon Cho, Seul Ki Kim, Shin-Hee Kim, Won Kyoung Cho, Min Ho Jung, Moon Bae Ahn

**Affiliations:** Department of Pediatrics, College of Medicine, Catholic University of Korea, Seoul 06591, Korea; rebekahjs@hanmail.net (J.-S.S.); soon926@hanmail.net (K.S.C.); seulki12633@gmail.com (S.K.K.); tigger1018@naver.com (S.-H.K.); wendy626@catholic.ac.kr (W.K.C.); jmhpe@catholic.ac.kr (M.H.J.)

**Keywords:** diabetic nephropathy, *N*-acetyl-β-D-glucosaminidase, pediatric obesity

## Abstract

Children with diabetes, and particularly those with obesity, have poor glycemic control. They are thus at higher risk of early microvascular complications. Renal tubulointerstitial markers are integral to evaluating diabetic nephropathy. Various biomarkers have been proposed, but their role in the obese pediatric population is uncertain. We investigated renal injury markers in children with diabetes, according to obesity, and determined their role as early predictors of diabetic nephropathy. Fifty-three children and adolescents, diagnosed with either type 1 or 2 diabetes mellitus, and 43 control children, aged 7–18 years, were included. Clinical and laboratory characteristics, including six renal injury markers, were compared among subjects according to body mass index and presence of diabetes mellitus. Urine neutrophil gelatinase-associated lipocalin, kidney injury molecule-1, and *N*-acetyl-β-D-glucosaminidase (NAG) showed significant difference between controls and diabetic children, whereas urine NAG was the only biomarker that was significantly lower either in non-obese or obese controls as compared to diabetic children. Urine NGAL, KIM-1, and NAG showed significant correlations with both HbA1c and urine ACR, whereas only urine NAG was significantly correlated with HbA1c even when groups were subdivided based on the presence of either obesity or diabetes. After adjusting for age, sex, body mass index, duration of known diabetes, and urine albumin-to-creatinine ratio, HbA1c remained a significant risk factor for elevated urine NAG. Urine NAG could be a useful indicator of tubulointerstitial damage in children with diabetes in the pre-albuminuric state. Tighter glycemic control appears to be crucial for avoiding early progression to diabetic nephropathy.

## 1. Introduction

With increasing incidence, childhood diabetes mellitus (DM) has become a major public health issue. Maintaining strict glycemic control is key to early prevention of the development of the complications of this disease [1]. Diabetic nephropathy (DN) is one of the major diabetes-associated adverse outcomes, characterized by albuminuria, hypertension, and the deterioration of renal function [2]. Numerous biomarkers have been introduced to assess tubulointerstitial damage. Recent studies have focused on investigating the feasibility of using renal injury markers for the detection of DN at an early stage in order to initiate prompt treatment [3,4]. Microvascular complications are not typical in the earlier course of childhood diabetes, since these are long-term sequelae of poor glycemic control. Nevertheless, management may be difficult when DN proceeds to irreversible end-stage renal disease. Thus, routine screening for renal function should start as early as possible from the beginning of diagnosis of childhood DM.

Obesity in youth with DM is a serious concern, since it may later be complicated by not only type 2 DM (T2DM), and is also rising among children with type 1 DM (T1DM), and insulin resistance (IR) could be triggered by either type of DM [5]. Obesity is characterized by excessive accumulation of adipose tissue, to the extent that it might cause metabolic complications. It is defined as when the body mass index (BMI) of an adolescent or child > 2 years exceeds the 95th percentile for age and sex [6,7]. Obese children are more likely to become obese adults and are also at risk of early comorbidities, such as hypertension, dyslipidemia, DM, and cardiovascular disease. According to recent studies, childhood obesity was suggested as a risk factor of renal injury. BMI correlated strongly with increased risk of end-stage renal disease [8]. Moreover, various biomarkers have been studied to investigate the association between obesity-induced IR and renal injury [9].

A number of renal injury markers have been introduced to identify those at risk of renal complications at an early stage. Urine biomarkers, such as neutrophil gelatinase-associated lipocalin (NGAL), beta-2 microglobulin (β2-M), kidney injury molecule-1 (KIM-1), liver-type fatty acid-binding protein (L-FABP), osteopontin (OPN), and *N*-acetyl-β-D-glucosaminidase (NAG), have been extensively studied to detect DN among the pediatric population [10]. Several studies have shown that levels of certain biomarkers were significantly elevated, particularly in obese as compared to normoweight children [11,12,13,14]. Thus, it is likely that there is close crosstalk between renal injury markers and the pathophysiology of obesity. In addition, several biomarkers, such as NGAL and OPN, have been suggested as candidates associated with microalbuminuria, which also seems to be correlated with IR and chronic low-grade inflammation among obese children [15].

Nevertheless, a link between renal injury markers and childhood obesity remains unclear, and the underlying pathophysiology is poorly understood. We hypothesized that obese children with diabetes may be more likely to exhibit poor glycemic control and exposed to a higher risk of tubulo-interstitial damage. The effect of obesity on DN needs to be elucidated. Moreover, identifying a practical renal injury marker in such a population is crucial for preventing the occurrence of early-stage DN, as well as for determining the role of childhood obesity in diabetic complications. Therefore, in this study, we investigate urine biomarkers, including NGAL, β2-M, KIM-1, L-FABP, OPN, and NAG, among children and adolescents with diabetes, in relation to obesity status, and suggest the most practical biomarker for predicting early renal injury in the pre-albuminuric stage.

## 2. Experimental Section

### 2.1. Study Subjects

The present study was a case–control study including 53 children previously diagnosed with either type 1 or 2 diabetes, and 43 controls aged 9–19 years. Subjects were recruited from July to December 2020 at Bucheon and Seoul St. Mary’s Hospital. Subjects with T1DM were treated with subcutaneous insulin, whereas those with T2DM were treated with metformin, with or without subcutaneous insulin. Control subjects were identified as those who visited the endocrinology clinic for the purpose of growth assessment. None of the controls had been diagnosed with underlying renal, endocrinologic, or metabolic disorders. In addition, none of the children with diabetes had diabetes-associated renal, cardiovascular, gastroesophageal, or psychiatric comorbidities.

The Institutional Review Board of the Catholic University of Korea approved this study (XC21SIDI0103 11 August 2021) according to the principles embodied in the Declaration of Helsinki. All participants and their guardians provided written informed consent.

### 2.2. Definition of Type 1 and 2 DM

Fifty-three children with diabetes met all of the following diagnostic criteria at initial presentation: (i) fasting blood glucose > 200 mg/mL, (ii) fasting blood glucose > 126 mg/mL, and (iii) glycosylated hemoglobin (HbA1c) > 6.5% with classic manifestation of polyuria, polydipsia, or weight loss [16]. Children with diabetes were classified as T1DM in the presence of more than one of the following anti-pancreatic autoantibodies: glutamic acid decarboxylase 65, tyrosine phosphatase-like insulinoma antigen 2, insulin, and β-cell-specific zinc transporter 8 autoantibodies. The remainder, who did not meet the criteria of T1DM, were classified as T2DM. Children who had a family history of DM of more than three generations underwent targeted next-generation sequencing; however, none were diagnosed with monogenic DM.

### 2.3. Clinical Data and Blood Sample Collection

For anthropometric measurements, height was measured using a Harpenden Stadiometer (Holtain^®^, Crymych, UK) and weight with a Simple Weighing Scale (Cas^®^, Seoul, Korea) at the time of blood collection. BMI (kg/m^2^) was calculated and then converted to the age- and sex-matched standard deviation scores (SDS) according to the national growth chart, whereas obese children were defined for BMI SDS ≥ 95th percentiles, or otherwise categorized as normoweight [17].

Fasting plasma samples were collected from children during visits to the outpatient clinic and included glucose, creatinine (Cr) aspartate aminotransferase (AST), alanine aminotransferase (ALT), C-reactive protein (CRP), total cholesterol, triglycerides, high-density lipoprotein cholesterols, low-density lipoprotein cholesterols, uric acid, HbA1c, C-peptide, and insulin. Additionally, the estimated glomerular filtration rate (eGFR) was calculated by using the revised Schwartz formula: 0.413 × height (cm)/plasma Cr (mg/dL) [18]. The homeostatic model assessment of insulin resistance (HOMA-IR) and β cell function (HOMA-β) were calculated by using the following formulas: fasting insulin (mU/L) × glucose (mg/dl)/405 and (360 × fasting insulin [mU/L]/(glucose [mg/dL]-63) [19].

### 2.4. Renal Injury Marker Measurements

Morning urine samples were collected and centrifuged at 3000× *g* at 4 °C for 15 min (U-32012 Centrifuge, Boeco^®^, Hamburg, Germany) followed by storage at −80 °C until assayed. The concentrations of renal injury markers were determined using commercially available enzyme-linked immunosorbent assays (NGAL, Human Lipocalin-2/NGAL Immunoassay, R&D Systems^®^, Minneapolis, MN, USA; β2-M, Human β2-Microglobulin Assay, R&D Systems^®^; KIM-1, Human TIM-1/KIM-1/HAVCR Immunoassay, R&D Systems^®^; L-FABP, Human FABP1/L-FABP DuoSet ELISA, R&D Systems^®^; OPN, Human Osteopontin Quantikine Kit, R&D Systems^®^; NAG, Human N-acetyl- β-D-glucosaminidase, NAG ELISA Kit, CUSABIO^®^, Wuhan, Hubei, China) in accordance with the manufacturer’s instructions and pre-test recommended dilution factor provided by individual assay kits. A microplate reader (SpectraMax 190 and SoftMax Pro7.0.2, Molecular Devices^®^, San Jose, CA, USA) was used to determine the concentrations. All samples were run in duplicate. The average coefficients of variation for urine intra-assay (inter-assay) precisions were 4.0% (6.7%), 5.9% (9.5%), 3.0% (6.2%), 6.0% (8.0%), 4.0% (6.6%), and <12% (<15%) for NGAL, β2-M, KIM-1, L-FABP, OPN, and NAG, respectively. A few were under the detectable range; therefore, concentrations were approximated to the lowest measurement of the population. Several concentrations were predicted based on the trend of the standard curve if not completely set within the standard range. All urine biomarkers as well as urine albumin concentrations were adjusted to the urine Cr concentration.

### 2.5. Statistical Analysis

For all descriptive variables, the normality of data distribution was determined by using the Shapiro–Wilk test. Since all variables were non-parametric, two subcategories were compared by means of the Mann–Whitney U test, whereas three or more groups were compared by the Kruskal–Wallis test. Spearman’s rank correlation was used to examine the correlations between 6 renal injury markers with clinical and biochemical data of diabetic children. Subsequently, multivariate regression analyses with HbA1c as the dependent variable were performed to determine the odds ratio (ORs) and 95% confidence intervals (CIs) and to analyze its association with renal injury markers after adjusting for age, sex, duration of known diabetes, BMI, and urine albumin-to-creatinine ratio (ACR). All statistical analyses were performed using SPSS version 24.0 (IBM Corp.^®^, Armonk, NY, USA).

## 3. Results

### 3.1. Baseline Characteristics of Study Subjects

This was a case–control study that included 53 children with diabetes (31 T1DM and 22 T2DM) and 43 controls enrolled by two institutions, and baseline clinical characteristics are described in Table 1. The median age of all subjects was 12.42 years (25th, 75th percentile interquartile range (IQR): 9.38, 15.38, respectively) and the duration of known diabetes was 2 years (0.75, 4.5) and 1.3 years (1.13, 2.85) for types 1 and 2 diabetic children, respectively. The prevalence of obesity was 60.5%, 22.6%, and 86.4% for controls, type 1, and type 2 diabetic children, respectively. The BMI SDS was not significantly different among the three obese groups, whereas the BMI SDS of obese type 2 diabetic children was the highest of all the study subjects. The number of non-obese controls was 17 (17.7%), with a median BMI SDS of 0.17 (−0.59, 0.99). Metabolic parameters, including plasma CRP, AST, and ALT levels, were higher in children with T2DM compared to controls. Plasma glucose, HbA1c, and HOMA-IR were elevated in children with T2DM as compared to controls, whereas C-peptide and HOMA-β were decreased in children with T1DM as compared to those with T2DM. The duration of known diabetes was not significantly different between type 1 and 2 diabetic children, whereas eGFR and urine ACR were not differed among the three groups.

### 3.2. Renal Injury Markers of Diabetic and Obese Children

**Figure 1:**
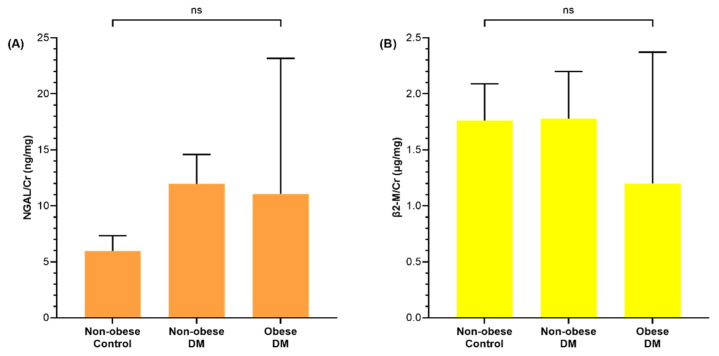

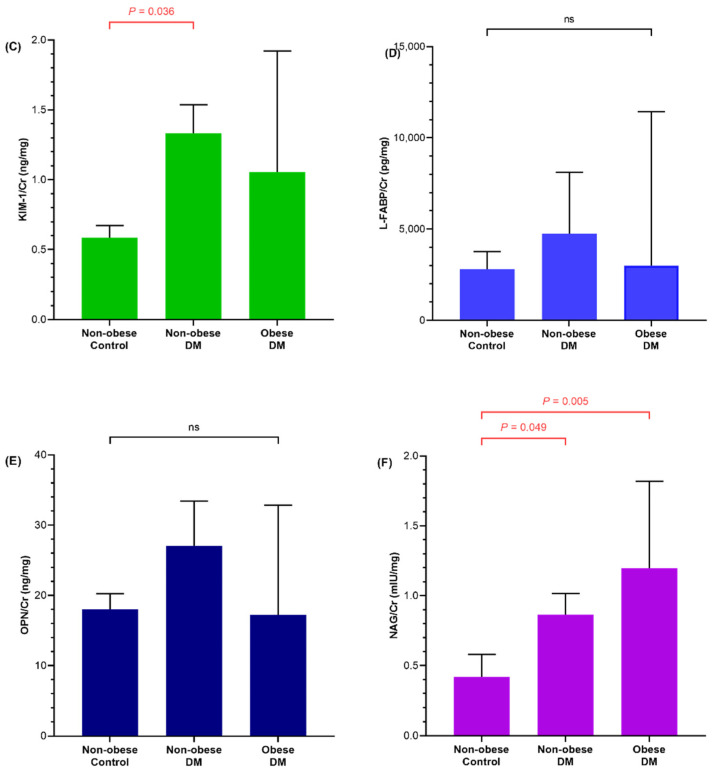
Bar graphs comparing urine (**A**) neutrophil gelatinase-associated lipocalin/creatinine (NGAL/Cr), (**B**) beta2-microglobulin/creatinine (β2-M/Cr), (**C**) kidney injury molecule-1/creatinine (KIM-1/Cr), (**D**) liver-type fatty acid-binding protein/creatinine (L-FABP/Cr), (**E**) osteopontin/creatinine (OPN/Cr), and (**F**) N-acetyl-D-glucosaminidase/creatinine (NAG/Cr) of non-obese control and diabetic children. Each bar and whisker indicates the mean value and standard error, respectively. The *p*-value is listed when it is significant, otherwise it is noted as non-specific (ns).

Urine NGAL, β2-M, KIM-1, L-FABP, OPN, and NAG levels were compared between diabetic children and non-obese controls and diabetic children (Figure 1). Urine NAG levels of non-obese (*p* = 0.049) and obese (*p* = 0.005) diabetic children as well as urine KIM-1 levels of non-obese (*p* = 0.036) diabetic children were significantly higher than non-obese controls, whereas urine NGAL, β2-M, L-FABP, and OPN levels showed no significant difference among the three groups.

**Figure 2:**
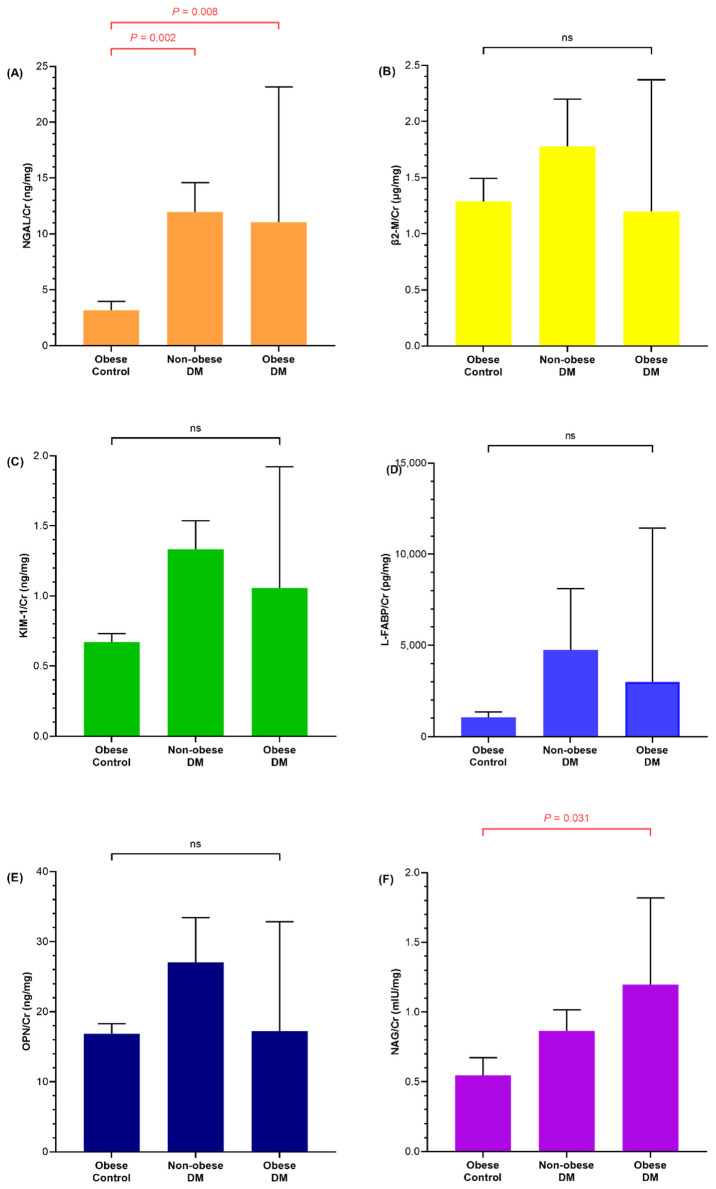
Bar graphs comparing urine (**A**) neutrophil gelatinase-associated lipocalin/creatinine (NGAL/Cr), (**B**) beta2-microglobulin/creatinine (β2-M/Cr), (**C**) kidney injury molecule-1/creatinine (KIM-1/Cr), (**D**) liver-type fatty acid-binding protein/creatinine (L-FABP/Cr), (**E**) osteopontin/creatinine (OPN/Cr), and (**F**) N-acetyl-D-glucosaminidase/creatinine (NAG/Cr) of obese control and diabetic children. Each bar and whisker indicates the mean value and standard error, respectively. The *p*-value is listed when it is significant, otherwise it is noted as non-specific (ns).

When the comparison was performed between obese controls and diabetic children, urine NGAL (*p* = 0.008) and NAG (*p* = 0.031) levels of obese diabetic children were significantly higher than those of obese controls (Figure 2). Urine NGAL and NAG levels of non-obese diabetic children were also higher than those of obese controls, and only NGAL (*p* = 0.002) was statistically significant.

**Figure 3:**
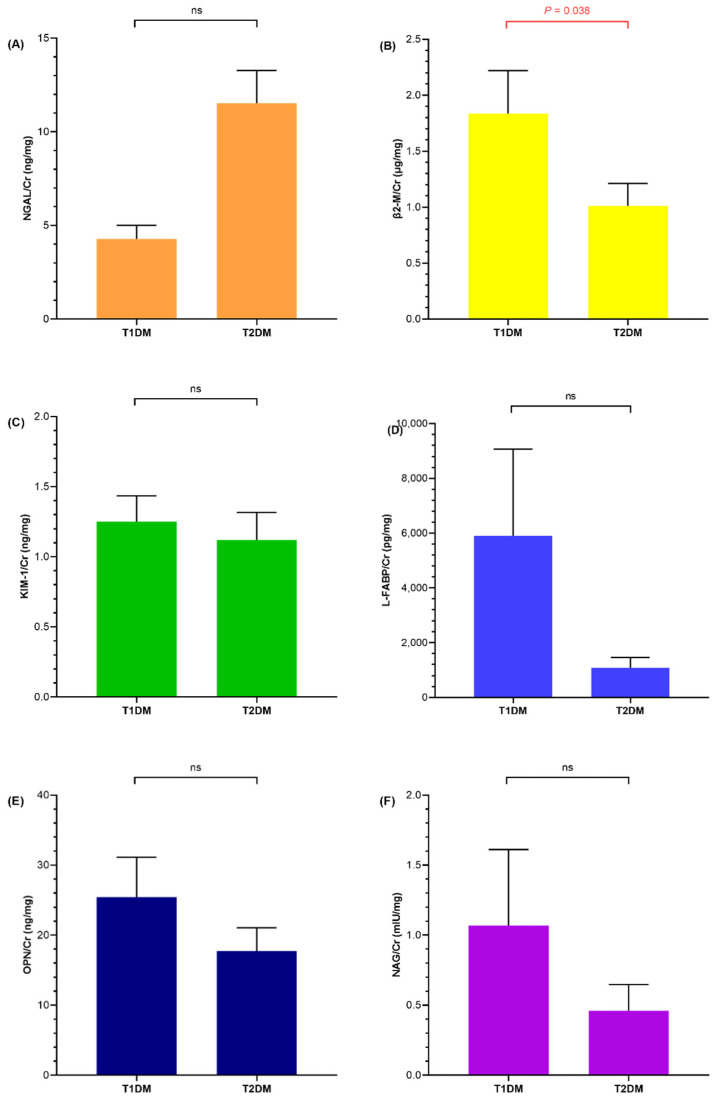
Bar graphs comparing urine (**A**) neutrophil gelatinase-associated lipocalin/creatinine (NGAL/Cr), (**B**) beta2-microglobulin/creatinine (β2-M/Cr), (**C**) kidney injury molecule-1/creatinine (KIM-1/Cr), (**D**) liver-type fatty acid-binding protein/creatinine (L-FABP/Cr), (**E**) osteopontin/creatinine (OPN/Cr), and (**F**) N-acetyl-D-glucosaminidase/creatinine (NAG/Cr) between children with type 1 diabetes mellitus (T1DM) and type 2 diabetes mellitus (T2DM). Each bar and whisker indicates the mean value and standard error, respectively. The *p*-value is listed when it is significant, otherwise it is noted as non-specific (ns).

When children with diabetes were subcategorized into T1DM and T2DM, the urine β2-M (*p* = 0.038) level was higher in children with T1DM than in children with T2DM, whereas the rest of renal injury markers showed no significant difference (Figure 3).

### 3.3. Correlation of Metabolic Parameters with Renal Injury Markers 

In Spearman correlation analyses, age, CRP, uric acid, HbA1c, c-peptide, HOMA-β, and urine ACR showed significant correlation with different renal injury markers (Table 2). Urine NGAL/Cr (rho = 0.21; *p* = 0.04), KIM-1/Cr (rho = 0.253; *p* = 0.013), and NAG/Cr (rho = 0.26; *p* = 0.01) correlated with HbA1c. HOMA-β correlated negatively with urine NGAL/Cr (rho = −0.3113; *p* = 0.002) and KIM-1/Cr (rho = −0.214; *p* = 0.036). All renal injury markers showed significant correlation with urine ACR; were positively correlated with urine NGAL/Cr (rho = 0.278; *p* = 0.006), KIM-1/Cr (rho = 0.22; *p* = 0.032), L-FABP/Cr (rho = 0.304; *p* = 0.003), OPN/Cr (rho = 0.217; *p* = 0.033), and NAG/Cr (rho = 0.438; *p* < 0.001); and were inversely correlated with urine β2-M (rho = −0.424; *p* < 0.001).

### 3.4. Association of Urine NGAL, KIM-1, and NAG with HbA1c

To identify the association of HbA1c with urine NGAL, KIM-1, and NAG of controls and diabetic children, Spearman correlation analyses were performed based on four groups (non-obese control, obese control, non-obese diabetic, and obese diabetic) (Table 3). The HbA1c of obese diabetic children showed significant correlation with urine NAG of non-obese controls (*p* = 0.033), non-obese (*p* = 0.018), and obese (*p* = 0.002) diabetic children, whereas the HbA1c of non-obese diabetic children was correlated with the urine NAG of non-obese (*p* = 0.004) and obese diabetic (*p* = 0.032) children. HbA1c showed no significant correlation with renal injury markers of both non-obese and obese controls except for urine KIM-1 of non-obese controls, which was correlated with HbA1c of obese controls (*p* = 0.005).

Multivariate regression analyses were performed using urine NGAL/Cr, KIM-1/Cr, and NAG/Cr as dependent variables (Table 4). The unadjusted OR for HbA1c, BMI, and urine ACR towards urine NAG/Cr elevation were 0.366 (95% CI = 0.074 to 0.658; *p* = 0.015), 0.076 (95% CI = −0.38 to 0.228; *p* = 0.617), and 6.713 (95 % CI = 0.3.471 to 9.955; *p* < 0.001), respectively. After adjustments made for age, sex, BMI, urine ACR, and duration of known diabetes, HbA1c (OR = 0.353; 95 % CI = 0.101–0.605; *p* = 0.007) remained a significant risk factor for urine NAG/Cr elevation. The unadjusted OR for urine ACR towards urine NGAL/Cr elevation was 19.69 (95 % CI = 0.379 to 93; *p* = 0.046); however, it was not significant towards urine KIM-1/Cr elevation (OR = 0.082; 95 % CI = −1.443 to 1.61; *p* = 0.915).

Furthermore, HbA1c was not associated with urine NGAL/Cr (OR = 0.228; 95% CI = −1.43 to 1.88; *p* = 0.784) or KIM-1/Cr elevation (OR = 0.019; 95 %CI = −0.106 to 0.145; *p* = 0.753). Adjustments for age, sex, BMI, urine ACR, and duration of diabetes did not affect the OR of HbA1c for either urine NGAL/Cr or KIM-1.

## 4. Discussion

Among urine NGAL, KIM-1, and NAG, which showed significant difference between controls and diabetic children, urine NAG was the only biomarker that was significantly lower either in non-obese or obese controls as compared to diabetic children. Urine NGAL, KIM-1, and NAG showed significant correlations with both HbA1c and urine ACR, whereas only urine NAG was significantly correlated with HbA1c, even when groups were subdivided based on the presence of either obesity or DM. Subsequently, higher HbA1c might be an important determinant that triggers urine NAG elevation in children with diabetes, regardless of age, sex, obesity, duration of known diabetes, and albuminuric status. Our findings suggested that urine NAG could be considered a more promising indicator for detecting the development of DN early in children with diabetes in a pre-albuminuric state than the other five renal injury markers investigated in our study. Obesity remains a strong environmental factor triggering the onset of DM, yet tighter glycemic control appears to be more critical than maintaining normal weight in order to reduce early progression to DN [20]. After exploring the characteristics of the six renal injury markers among obese and non-obese children and children with and without diabetes, we suggest that urine NAG could be a more sensitive biomarker that is closely linked to glycemic status, and thus could be considered an early indicator of future development of DN. To the best of our knowledge, no previous study had investigated six renal injury markers simultaneously between obese and non-obese children with diabetes in a pre-albuminuric state.

NAG is a 140-kDa lysosomal enzyme predominantly secreted by proximal tubules, and its elevation is found specifically upon renal injury, such as in primary glomerulonephritis and acute kidney injury [21]. During the earlier course of DN, impaired tubular function and structural alteration resulting in microalbuminuria are vital components. For many years, urine NAG has been used as a highly sensitive biomarker for the early detection of renal disease, regardless of albuminuric status, and is also considered a predictor for cardiovascular and hepatic complications of diabetes in adults [22,23,24]. The role of urine NAG in children with diabetes has not yet been clearly determined and further clinical evidence is needed. Habashy et al. reported that urine NAG, among several biomarkers for assessing DN, was considered to be the most predictable marker of microalbuminuria in children with T1DM, with high sensitivity, specificity, and diagnostic accuracy [25]. In another study by Mysilwiec et al., NAG enzyme activity was associated with the development and the degree of both DN and retinopathy in adults who were diagnosed with T1DM during the childhood and adolescence period [26]. In our study, even though urine NAG was associated with HbA1c of children with T1DM, the interpretation was limited, since none of the children with diabetes showed progression to DN, as evidenced by normal plasma Cr and urine ACR < 30 mg/g.

Controversy exists about the role of NAG among those who are obese. According to Goknar et al., urine NAG was significantly higher in obese than non-obese children, regardless of their glycemic state. However, it was not different between those with or without IR [14]. In another study by Safaeian et al., urine NAG was also higher in obese as compared to non-obese children, but no significant correlation existed between urine NAG and albumin excretion, implying that urine NAG might be a biomarker of renal damage in obese children [27]. In contrast, urine NAG of adults with T2DM correlated negatively with BMI, and it was suggested to be a sensitive biomarker of glucose fluctuation and insulin secretory capacity [28]. Moreover, the role of urine NAG in adults was more likely to predict obesity-induced metabolic conditions, such as cardiovascular disease and nonalcoholic fatty liver disease, rather than being directly linked to higher BMI status [24,28]. Nevertheless, the underlying pathophysiological mechanism associated with the elevation of urine NAG in both obesity and obesity-related metabolic conditions remains unknown. The urine NAG and BMI SDS in our study participants appeared to be inversely correlated in our children with T2DM, since their BMI SDS was significantly higher, whereas their urine NAG was lower as compared to controls. Nevertheless, urine NAG was not different between obese and non-obese children, indicating that the presence of DM might be more significantly associated with the urine NAG of obese children than with obesity status itself.

Tight glycemic control is a cornerstone in the prevention of diabetes-induced microvascular complications, including DN. HbA1c has been the most valuable indicator of glucose monitoring, and achieving A1c < 7% was suggested as a universal goal for any child with diabetes, in order to minimize the development of comorbidities [29]. Routine measurement of HbA1c and urine ACR is a widely recognized method of screening for early detection of DN among children with diabetes. The progression to DN could still occur in individuals in a normoalbuminuric state, since tubule-interstitial damage can precede glomerular injury [11]. Therefore, an ideal biomarker complementary to the role of HbA1c and urine ACR is needed, and the discovery of such a biomarker would help to identify early suspected tubule-interstitial injury during the pre-albuminuric stage, or before reduction of eGFR. Among all six renal injury markers that showed a significant correlation with urine ACR, only urine NGAL, KIM-1, and NAG were simultaneously correlated with HbA1c. However, urine NAG was the only renal injury marker that was significantly associated with the increase in HbA1c regardless of age, sex, obesity, and albuminuric status in our study. These findings implied that urine NAG could be a reliable indicator of the early DN progression of children with diabetes without being influenced by the degree of weight and albuminuric status. In addition, it also appeared to have a stronger association with glycemic status than did the other five renal injury markers.

Our study had a few limitations. First, this study was an age-unmatched case–control study conducted on a small sample population, within a cross-sectional design. Sample sizes for children with T1DM and T2DM were not equivalent, either. Although several renal injury marker levels showed significant differences between subcategorized groups, for each renal injury marker, evidenced-based pediatric reference intervals were lacking. Second, none of the children with diabetes had previously been diagnosed with DN or had increased plasma Cr and urine ACR > 30 mg/g. The correlation of renal injury markers with hyperglycemia and microalbuminuria might have been more clearly addressed if children with previously diagnosed DN were included. Third, the control group was non-diabetic, but not truly non-obese, as indicated by a BMI SDS as high as 2.31. In addition, the non-obese group included normoweight as well as over- and underweight children. This might have caused selection bias and influenced the comparison of renal injury markers between controls and children with diabetes. Nevertheless, to the best of our knowledge, no previous study had examined NGAL, β2-M, KIM-1, L-FABP, OPN, and NAG in children with diabetes and compared these levels with those of children without diabetes with respect to obesity. Our data could be valuable for future reference of these six renal injury markers in such populations, considering that these data remain scarce.

## 5. Conclusions

In conclusion, urine NAG could be an early indicator specific for tubulointerstitial damage in childhood diabetes in a pre-albuminuric state. It was also significantly associated with HbA1c elevation, regardless of age, sex, and albuminuria, as well as obesity status. Being overweight or obese did not affect the level of renal injury markers; however, tighter glycemic control appeared to be the key intervention to avoid early DN progression. Prospective studies in a larger sample size are needed to examine the role of renal injury markers among adolescents and children with diabetes and adolescents in relation to obesity status.

## Figures and Tables

**Table 1:** Clinical and biochemical characteristics of the study subjects.

	Total (*n* = 96)						
	Control (*n* = 43)		T1DM (*n* = 31)		T2DM (*n* = 22)		*p*
	Non-Obese (*n* = 17)	Obese (*n* = 26)	Non-Obese (*n* = 24)	Obese (*n* = 7)	Non-Obese (*n* = 3)	Obese (*n* = 19)	
**Age (years)**	8.83 (8.54, 10.63)	10.54 (9.42, 12.04)	14 (9.96, 16.48)	15 (12.5, 15.83)	16.58 (15.83, 16.92)	17.83 (15.75, 18.33)	<0.001
**^†^** **Sex**	7/10	20/6	14/10	4/3	0/3	10/9	0.075
**^‡^** **Institution**	0/17	0/26	15/9	2/5	2/1	11/8	<0.001
**Height SDS**	−0.91 (−1.35, 0.39)	1.23 (0.4, 1.61)	0.2 (−0.39. 0.81)	0.26 (−0.78, 1.57)	1.81 (−0.88, 2.26)	0.84 (0.07, 1.81)	<0.001
**Weight SDS**	−0.09 (−0.92, 0.48)	2.46 (2.14, 2.99)	−0.33 (−1.05, 0.42)	234 (1.47, 2.99)	0.19 (0.14, 1.34)	3.38 (2.44, 4.26)	<0.001
**BMI SDS**	0.17 (−0.59, 0.99)	2.73 (2.46, 3.24)	−0.68 (−1.44, 0.44)	2.43 (1.84, 2.93)	0.31 (−0.77, 0.64)	3.26 (2.88, 4.79)	<0.001
**CRP (mg/dL)**	0.03 (0.03, 0.11)	0.1 (0.05, 0.19)	0.3 (0.03, 0.3)	1.32 (0.25, 3.93)	1.02 (0.03, 3.39)	0.73 (0.12, 2.6)	<0.001
**Glucose (mg/dL)**	94.5 (90, 97.75)	94 (91, 97.5)	109 (100.25, 140)	224 (161, 248)	113 (90, 120)	138 (112, 254)	0.284
**Cr (mg/dL)**	0.46 (0.42, 0.49)	0.51 (0.44, 0.61)	0.54 (0.44, 0.71)	0.48 (0.45, 0.57)	0.54 (0.53, 0.6)	0.69 (0.55, 0.81)	0.003
**eGFR (mL/min/1.73 m^2^)**	119.32 (107.55, 127.56)	121.62 (104.98, 131.67)	113.87 (98.35, 131.79)	138.79 (123.9, 143.09)	121.33 (116.6, 131.7)	103.25 (89.89, 133.05)	<0.001
**AST (U/L)**	25 (21, 28.75)	22 (18, 28)	17 (13.25, 18.75)	21 (16, 27)	15 (8, 37)	34 (13, 59)	0.611
**ALT (U/L)**	14 (9.5, 16.75)	18 (13, 41)	10.5 (9, 13.75)	17 (13, 25)	10 (4, 65)	57 (16, 103)	0.005
**TC (mg/dL)**	182.5 (152.25, 225)	170 (156.5, 196)	167.5 (148.25, 197.75)	204 (159, 213)	193 (177, 207)	181 (159, 202)	<0.001
**TG (mg/dL)**	97.5 (71, 156.75)	98 (56, 129.5)	69 (45.25, 111.75)	174 (109, 290)	111 (99, 164)	127 (92, 238)	0.237
**HDL-C (mg/dL)**	54.5 (44, 64.75)	49 (44, 55.5)	60 (52, 70)	58 (48, 72)	50 (47, 56)	44 (39, 46)	<0.001
**LDL-C (mg/dL)**	108.5 (87.5, 137)	104 (95.5, 128)	87.8 (74.8, 125.05)	98.8 (72.2, 124)	123.2 (122, 127.2)	113.6 (90, 122.2)	<0.001
**Uric acid (mg/dL)**	4.3 (3.7, 4.95)	6.35 (4.88, 7.13)	4.35 (3.58, 5.28)	4.5 (4, 6)	4.4 (2.4, 5.7)	5.5 (4.9, 6.5)	<0.001
**HbA1c (%)**	5.2 (5.05, 5.45)	5.5 (5.38, 5.7)	7.8 (6.55, 9.18)	9.5 (8.5, 10.3)	7.4 (6.8, 7.9)	8.4 (5.9, 11.7)	<0.001
**C-peptide (ng/mL)**	1.91 (1.48, 2.77)	2.63 (2.18, 3.79)	0.15 (0.02, 0.6)	0.11 (0.02, 0.9)	1.46 (0.94, 4.74)	3.26 (2.04, 4.57)	<0.001
**HOMA-IR**	2.43 (1.73, 5.23)	4.79 (2.73, 6.5)	0.74 (0.36, 1.38)	1.94 (1.62, 11.06)	1.23 (1.22, 8.68)	6.59 (4.39, 12.84)	<0.001
**HOMA-β (%)**	110.45 (87.06, 246.86)	229.24 (171.4, 299.24)	23.83 (9.58, 48.41)	16.79 (6.17, 26.54)	31.75 (26.02, 520.53)	120.26 (67.68, 209.39)	<0.001
**Urine ACR (mg/g)**	8.03 (4.14, 12)	4.12 (3.43, 5.48)	8.48 (6.54, 13.44)	20.2 (6.51, 193.43)	18.5 (4.96, 41.09)	11.69 (6.99, 25.32)	0.012

Values are expressed as median (interquartile range 25%, 75 %). ^†^ Male/female. ^‡^ Bucheon St. Mary’s Hospital/Seoul St. Mary’s Hospital. All measurements are plasma values unless otherwise noted. ACR, albumin-to-creatinine ratio; ALT, alanine aminotransferase; AST, aspartate aminotransferase; BMI SDS, body mass index standard deviation score; Cr, creatinine; CRP, C-reactive protein; DM, diabetes mellitus; eGFR, estimated glomerular filtration rate; HbA1c, glycosylated hemoglobin; HDL-C, high-density lipoprotein cholesterol; HOMA-IR, homeostatic model assessment of insulin resistance; HOMA-β, homeostatic model assessment of beta cell function; LDL-C, low-density lipoprotein cholesterol; T1DM, type 1 diabetes mellitus; T2DM, type 2 diabetes mellitus; TC, total cholesterol; TG, triglycerides.

**Table 2:** The correlation of 6 renal injury markers with clinical and biochemical parameters of study subjects.

	NGAL		β2-M		KIM−1		L-FABP		OPN		NAG	
	rho	*p*	rho	*p*	rho	*p*	rho	*p*	rho	*p*	rho	*p*
**Age**	0.116	0.259	**−0.369**	**<0.001**	0.015	0.885	**−0.296**	**0.003**	−0.135	0.19	**−0.398**	**<0.001**
**BMI SDS**	−0.094	0.363	−0.166	0.106	−0.019	0.854	−0.058	0.573	−0.16	0.12	−0.137	0.182
**CRP**	0.193	0.059	−0.072	0.489	0.104	0.312	−0.068	0.509	−0.047	0.65	**−0.264**	**0.009**
**AST**	−0.042	0.687	0.106	0.305	−0.108	0.293	0.065	0.529	−0.15	0.143	0.062	0.546
**ALT**	−0.108	0.297	−0.011	0.915	−0.094	0.36	−0.071	0.493	−0.187	0.068	−0.066	0.523
**TC**	0.103	0.317	0.075	0.465	0.144	0.163	0.019	0.853	0.041	0.694	0.155	0.13
**TG**	0.123	0.233	−0.005	0.962	−0.064	0.537	−0.008	0.939	−0.059	0.565	0.074	0.472
**HDL-C**	0.043	0.676	0.058	0.573	−0.007	0.946	0.061	0.554	−0.006	0.951	−0.064	0.534
**LDL-C**	0.023	0.827	−0.025	0.808	0.172	0.094	−0.057	0.584	0.004	0.965	0.102	0.321
**Uric acid**	**−0.24**	**0.018**	−0.2	0.051	−0.099	0.335	−0.195	0.057	−0.044	0.672	−0.132	0.2
**HbA1c**	**0.21**	**0.04**	−0.1	0.332	**0.253**	**0.013**	−0.027	0.797	−0.072	0.484	**0.26**	**0.01**
**C-peptide**	**−0.241**	**0.018**	−0.155	0.131	−0.103	0.318	−0.103	0.318	−0.045	0.662	−0.038	0.712
**HOMA-IR**	−0.159	0.122	−0.095	0.356	−0.034	0.745	−0.053	0.608	0.007	0.948	0.015	0.881
**HOMA-β**	**−0.313**	**0.002**	−0.066	0.521	**−0.214**	**0.036**	−0.057	0.583	0.017	0.869	−0.04	0.701
**Urine ACR**	**0.278**	**0.006**	**−0.424**	**<0.001**	**0.22**	**0.032**	**0.304**	**0.003**	**0.217**	**0.033**	**0.438**	**<0.001**

All renal injury markers are adjusted to urine creatinine. ACR, albumin-to-creatinine ratio; ALT, alanine aminotransferase; AST, aspartate aminotransferase; β2-M, beta-2 microglobulin; BMI SDS, body mass index standard deviation score; CRP, C-reactive protein; HbA1c, glycosylated hemoglobin; HDL-C, high-density lipoprotein cholesterol; HOMA-IR, homeostatic model assessment of insulin resistance; HOMA-β, homeostatic model assessment of beta cell function; KIM-1, kidney injury molecule-1; L-FABP, liver-type fatty acid-binding protein; LDL-C, low-density lipoprotein cholesterol; NAG, *N*-acetyl-β-D-glucosaminidase; NGAL, neutrophil gelatinase-associated lipocalin; OPN, osteopontin; TC, total cholesterol; TG, triglycerides.

**Table 3:** The correlation of urine neutrophil gelatinase-associated lipocalin (NGAL), kidney injury molecule-1 (KIM-1), and N-acetyl-D-glucosaminidase (NAG) with glycosylated hemoglobin (HbA1c) of study subjects with and without obesity and diabetes mellitus (DM).

		HbA1c							
		Non-Obese Control		Obese Control		Non-Obese Diabetic		Obese Diabetic	
		rho	*p*	rho	*p*	rho	*p*	rho	*p*
**NGAL**	**Non-obese control**	−0.044	0.868	0.072	0.784	0.113	0.666	0.27	0.294
	**Obese control**	0.115	0.661	−0.116	0.574	−0.135	0.512	0.166	0.417
	**Non-obese diabetic**	−0.13	0.62	0.108	0.601	−0.081	0.687	−0.423	0.031
	**Obese diabetic**	0.029	0.913	−0.06	0.772	0.193	0.346	0.267	0.187
**KIM−1**	**Non-obese control**	−0.231	0.373	**0.647**	**0.005**	0.322	0.208	0.140	0.592
	**Obese control**	0.052	0.842	−0.248	0.222	0.119	0.561	0.159	0.438
	**Non-obese diabetic**	0.277	0.282	0.069	0.738	−0.125	0.533	−0.294	0.145
	**Obese diabetic**	−0.439	0.078	−0.152	0.459	0.125	0.543	0.279	0.168
**NAG**	**Non-obese control**	−0.029	0.913	0.041	0.841	0.228	0.262	**0.419**	**0.033**
	**Obese control**	−0.121	0.644	0.058	0.779	0.259	0.201	0.032	0.877
	**Non-obese diabetic**	0.108	0.679	0.346	0.173	**0.143**	**0.004**	**0.056**	**0.018**
	**Obese diabetic**	−0.175	0.503	−0.189	0.355	**0.105**	**0.032**	**0.096**	**0.002**

All renal injury markers are adjusted to urine creatinine. HbA1c, glycosylated hemoglobin; HDL-C, high-density lipoprotein cholesterol; HOMA-IR, homeostatic model assessment of insulin resistance; HOMA-β, homeostatic model assessment of beta cell function; LDL-C, low-density lipoprotein cholesterol; TC, total cholesterol; TG, triglycerides.

**Table 4:** Multivariate regression analyses of glycosylated hemoglobin (HbA1c) with increased urine neutrophil gelatinase-associated lipocalin (NGAL), kidney injury molecule-1 (KIM-1), and N-acetyl-D-glucosaminidase (NAG) of diabetic children.

	NGAL			KIM-1			NAG		
HbA1c	OR (95% CI)	SE	*p*	OR (95% CI)	SE	*p*	OR (95% CI)	SE	*p*
**Unadjusted**	0.228 (−1.43–1.88)	0.825	0.784	0.019 (−0.106–0.145)	0.063	0.753	**0.366 (0.074–0.658)**	**0.145**	**0.015**
**Adjusted**									
**Model 1**	0.631 (−0.899–2.16)	0.76	0.411	0.035 (−0.086–0.156)	0.06	0.56	**0.355 (0.081–0.629)**	**0.136**	**0.012**
**Model 2**	0.522 (−1.08–2.13)	0.797	0.516	0.057 (−0.068–0.182)	0.062	0.363	**0.378 (0.091–0.665)**	**0.143**	**0.011**
**Model 3**	0.442 (−112–2)	0.772	0.569	0.056 (−0.07–0.183)	0.063	0.375	**0.353 (0.101–0.605)**	**0.125**	**0.007**

Urine NGAL, KIM-1, and NAG were adjusted to urine creatinine. Model 1: adjusted for age, sex and duration of known diabetes; model 2: adjusted for age, sex, duration of known diabetes and body mass index; model 3: adjusted for age, sex, duration of known diabetes, body mass index, and urine albumin-to-creatinine ratio CI, confidence interval; OR, odds ratio; SE, standard error.

## Data Availability

The raw data supporting the conclusions of this article will be made available by the authors, without undue reservation.
